# Broca's area processes the hierarchical organization of observed action

**DOI:** 10.3389/fnhum.2013.00937

**Published:** 2014-01-17

**Authors:** Masumi Wakita

**Affiliations:** Department of Behavioral and Brain Sciences, Primate Research Institute, Kyoto UniversityInuyama, Japan

**Keywords:** near-infrared spectroscopy, Broca's area, language, music, action, hierarchy, syntax

## Abstract

Broca's area has been suggested as the area responsible for the domain-general hierarchical processing of language and music. Although meaningful action shares a common hierarchical structure with language and music, the role of Broca's area in this domain remains controversial. To address the involvement of Broca's area in the processing action hierarchy, the activation of Broca's area was measured using near-infrared spectroscopy. Measurements were taken while participants watched silent movies that featured hand movements playing familiar and unfamiliar melodies. The unfamiliar melodies were reversed versions of the familiar melodies. Additionally, to investigate the effect of a motor experience on the activation of Broca's area, the participants were divided into well-trained and less-trained groups. The results showed that Broca's area in the well-trained participants demonstrated a significantly larger activation in response to the hand motion when an unfamiliar melody was played than when a familiar melody was played. However, Broca's area in the less-trained participants did not show a contrast between conditions despite identical abilities of the two participant groups to identify the melodies by watching key pressing actions. These results are consistent with previous findings that Broca's area exhibits increased activation in response to grammatically violated sentences and musically deviated chord progressions as well as the finding that this region does not represent the processing of grammatical structure in less-proficient foreign language speakers. Thus, the current study suggests that Broca's area represents action hierarchy and that sufficiently long motor training is necessary for it to become sensitive to motor syntax. Therefore, the notion that hierarchical processing in Broca's area is a common function shared between language and music may help to explain the role of Broca's area in action perception.

## Introduction

Human language consists of a hierarchical structure in which phonemes are combined to form words, phrases and sentences up to the discourse level of speech structure according to several levels of rules. Uchiyama et al. (2008) studied brain regions involved in the structural analysis of language and revealed a posterior–anterior functional gradient with substantial overlap in the left inferior frontal region: phonological processes are localized in the Brodmann area (BA) 44, the processing of sentence structure is localized in BA 45 and semantic processing at the sentence level is related to BA 47 activity (also see Friederici and Kotz, 2003; Hagoort, 2005).

In the case of music, similarly to language, combinations of sequential and simultaneous notes make rhythms, melodies and harmonies to form an overall musical structure according to rules such as chord progressions. There is also a posterior-anterior functional gradient in the inferior frontal region in relation to the processing of musical hierarchy. For instance, the activation of BA 6/44 for rhythm discrimination and melody matching may reflect the processing of sequential sounds (Platel et al., 1997; Brown and Martinez, 2007). BA 44/45 is involved in harmonic evaluation (Tillmann et al., 2003; Brown and Martinez, 2007). Furthermore, BA 47 is sensitive to the meaning (or impression) of melody, reflecting the semantic processing of music (Platel et al., 1997). The similarities of linguistic and musical analysis raise the possibility that hierarchical processing in 2 different domains is subserved by common neural resources such as Broca's area (BAs 44 and 45) (Maess et al., 2001; Koelsch et al., 2002; Patel, 2003; Brown et al., 2006; Schön and François, 2011).

Action, as well as language and music, is organized hierarchically. A chain of individually meaningless body movements (such as extending an arm and opening a palm) can be combined into units of actions (such as reaching and grasping). These units of actions can be integrated into meaningful behavior (such as picking up a peanut). Despite evidence for the possible contribution of Broca's area in the hierarchical processing of observed skilled action (Fiebach and Schubotz, 2006; Tettamanti and Weniger, 2006; Fadiga et al., 2009; Higuchi et al., 2009; Wakita and Hiraishi, 2011), few studies have directly examined such involvement.

For instance, Higuchi et al. (2009) found an overlap of activity between tasks related to language and tool-use action in Broca's area. The authors suggested a domain-general role of Broca's area in hierarchical processing since language and tool-use action share computational principles for processing hierarchical structures common to these two domains. However, they did not directly test the involvement of Broca's area in the processing of action hierarchy. In order to confirm such an involvement, it is necessary to study whether Broca's area is sensitive to the degree of hierarchical complexity or to the sequential order of action.

Thus far, several patterns of musical action have been adopted to study the involvement of Broca's area in the hierarchical processing of observed action. Musical action consists of hierarchical structures in that the visual processing of structured key-press sequences and the integration of this information with the location of the fingers on the piano keyboard are essential to correctly identify harmony or melody by observing silent hand motion (Hasegawa et al., 2004).

For instance, Sammler et al. (2013) conducted an electroencephalography (EEG) study in which expert pianists watched silent videos of a hand playing either congruent or incongruent 5-chord sequences. The video corresponded to a linguistic task that tested the grammatical sensitivity of Broca's area. The results of the study revealed a higher activation of this area in response to sentences with non-canonical word order compared with sentences with standard word order (Friederici et al., 2006). The authors found positivity that possibly originated from a left inferior frontal source when participants observed hand trajectory toward syntactically incongruent chords. These findings indicate that the syntactical analysis of observed action is represented in Broca's area. However, such a silent harmony abstraction task is difficult to perform because theoretical knowledge of western harmony is necessary. Therefore, participation in this task is restricted to professional musicians.

In another study, using functional magnetic resonance imaging (fMRI), non-musicians watched silent videos of hands playing the piano while trying to identify the melodies (Hasegawa et al., 2004). This task may measure the sensitivity of Broca's area to tone or phoneme segmentation and reveal the activation of Broca's area to learned regularity (Abla and Okanoya, 2008; Karuza et al., 2013). Because less-trained participants could identify some melodies solely through the observation of silent key-touching sequences, this task may be adaptable to a larger number of participants than the silent harmony abstraction task. The authors' target region was the left planum temporale, but they observed higher levels of activation of BA 44 in well-trained participants compared with less-trained and naïve participants when they watched silent videos of hands playing melodies. However, in general, melody identification performance in this study was not good, even among the well-trained participants. Thus, the activation of Broca's area may have reflected an embodied simulation of piano playing rather than hierarchical processing.

In this study, to test whether Broca's area is involved in processing the hierarchical organization of observed action, well-trained and less-trained participants watched silent movies that featured hand movements playing familiar and unfamiliar melodies. The activation of Broca's area was measured using near-infrared spectroscopy (NIRS). The NIRS results for familiar and unfamiliar melodies were compared to reveal the involvement of Broca's area in the hierarchical analysis of observed action. Additionally, the comparison of NIRS results between well-trained and less-trained participants was examined to determine the influence of skill in the observed action on the activation of Broca's area.

All participants in the current study had sufficient knowledge about the familiar melodies preceding the experiments. Thus, tracking hand motions to identify the familiar melodies and even anticipating forthcoming action sequences may be easy. However, no participant had knowledge about the unfamiliar melodies. Thus, the participants had to pay extra attention to the hand motions to detect musical segments from stimulus movies and integrate them into whole melodies. Therefore, if a greater activation of Broca's area was found for unfamiliar than familiar melodies, it could be deduced that this area plays an essential role in the hierarchical processing of an observed action.

Although NIRS cannot detect deep brain activity, this technique offers several advantages. NIRS detects changes in hemoglobin concentration associated with brain activation occurring beneath the optodes. Therefore, this technique enables the estimation of the activation source using fewer sensors than are required for EEG. Additionally, NIRS allows the participants to maintain a comfortable posture in a quiet room during measurement, while body movements of participants are strictly restricted during an fMRI session. Thus, NIRS is a suitable technique to measure activity in Broca's area and is advantageous, especially for the participants.

## Methods

### Subjects

The subjects were 20 healthy, right-handed adults aged 23–40 years. Handedness scores were determined using the Edinburgh handedness inventory (Oldfield, 1971). The subjects were divided into 2 groups according to the criterion by Hasegawa et al. (2004). The subjects in the well-trained group (10 females; mean age, 28.7 years; range, 23–34 years) had received formal piano lessons for at least 8 years (mean, 11.4 years; range, 8–16 years). The subjects in the less-trained group (2 males and 8 females; mean age, 29.9 years; range, 24–40 years) had received formal piano lessons for less than 8 years (mean, 3.4 years; range, 1–7 years). All subjects were able to identify the familiar melodies by watching the key-touching hand movements. Prior to the experiment, the subjects were informed about the nature of the experimental procedures and gave their written informed consent for the study. This study was approved by the ethics committee of the Primate Research Institute, Kyoto University.

### Stimulus

**Figure 1:**
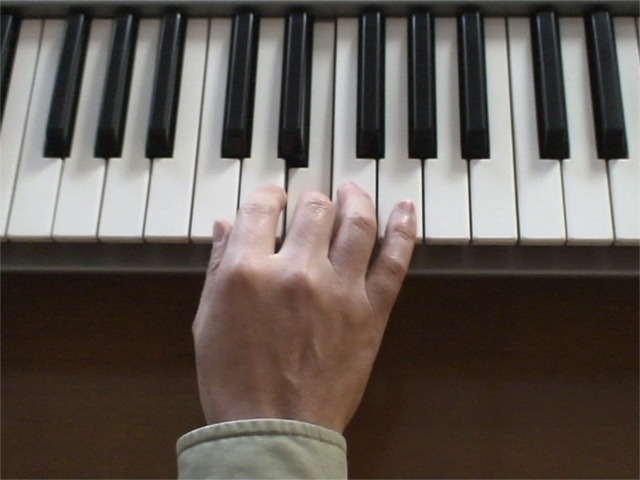
**Static example of a stimulus movie**.

First, hand movements playing the familiar melodies “Mary Had a Little Lamb” (M) and “London Bridge is Falling Down” (L) (key, C major; tempo, 120 beats per minute; duration, 8 s) on the keyboard were filmed (640 × 480 pixels) (Figure 1). There were fade-in and fade-out periods (1 s) in the movies to prevent the subjects from using the initial hand position as a cue to identify the melody. By making temporally reversed versions of these movies, “musically” unfamiliar stimuli were created. Consequently, four movies (familiar M and L movies and unfamiliar M and L movies) were generated. Two identical movies were combined to make the 16-s stimuli. No auditory signal was included in the stimuli (supplementary videos). Stimuli were presented on a 17-inch liquid crystal display monitor placed ~70 cm from the subjects' heads.

The unfamiliar stimuli contained spatial and temporal distributions of key-press durations of hand movement that were identical to those of the familiar stimuli. However, by reversing the temporal order, the sequential structure of hand motions (corresponding to note transitions) was rendered largely different between familiar and unfamiliar stimuli.

## Procedure

### Action observation task

An experimental session consisted of 12 trials. Each trial lasted 16 s, during which one of the four stimuli was presented. The length of the inter-trial interval was 25 s. All four movies were presented three times in a pseudorandom order, with each session beginning with a familiar stimulus. Every stimulus was presented once in four successive trials, and the same melody (M and L) and/or familiarity (familiar and unfamiliar) condition was repeated in no more than 3 consecutive trials. The participants were instructed to watch the movies carefully to identify the “melody” performed by the hand. The beginning of each stimulus movie was accompanied by a tone pip. The participants were asked to open their eyes gently when they heard the tone and close their eyes gently when the stimulus movie disappeared. Thus, the individuals' eyes were closed during the rest period. The entire experiment lasted for ~30 min.

Prior to the beginning of the recording session, the participants practiced for 8 trials (4 stimuli × 2 trials) with experimental stimuli to confirm that they understood the instructions.

### Near-infrared spectroscopy measurements

Cortical activity was continuously recorded throughout the experiment. Relative changes in oxy-hemoglobin (oxy-Hb) concentrations were measured using the NIRS system (ETG-100, Hitachi Medical Corporation, Tokyo, Japan). The sampling rate was set to 10 Hz. Nine optodes (in a lattice pattern forming 12 channels) were placed on both hemispheres. Because the main region of interest was Broca's area, a single recording channel was determined as a point between the optode nearest to F7 of the international 10–20 system and its posteriorly adjacent optode. Thus, NIRS results presumably reflected activity from Broca's area since F7 on the scalp has been reported to project onto the cortical surface of the anterior portion of the inferior frontal cortex (BA 45/47) (Okamoto et al., 2004, 2006; Koessler et al., 2009). Typically, the position of the target channel was located ~3 cm above the level of the supraorbital process and ~3 cm posterior to the lateral margin of the orbit.

It is true that cranio-cerebral correspondence using the international 10–20 system has been suggested (Okamoto et al., 2004, 2006; Koessler et al., 2009). However, there is substantial variation in the precise location and topographic extent of Broca's area among individuals (Amunts et al., 1999). Therefore, I acknowledge the difficulty in ensuring the degree of contribution of different subdivisions within the inferior frontal cortex to the NIRS signal.

The cortical responses for each trial were stimulus-locked and extracted from the raw oxy-Hb time series data. Pulsatile fluctuations were removed from the results by smoothing the oxy-Hb time series backward in time with a 5-s moving window. Baseline drift was corrected using linear interpolation between the time point of task onset and the time point of stimulus onset of the next trial. The oxy-Hb time series data from familiar and unfamiliar trials were individually averaged over trials, independent of the melody types. Finally, a mean oxy-Hb value per time point within a peristimulus period (a 16-s time window from 5 s after stimulus onset to 5 s after stimulus offset) was calculated for each condition. The averaged oxy-Hb values obtained during the familiar and unfamiliar conditions were then submitted to a paired *t*-test to assess the involvement of Broca's area in the hierarchical processing of observed action. The significance level was set to 5%.

One may assume that the degree of involvement of Broca's area to action hierarchy is influenced by the experience. Therefore, the relationship between this involvement of Broca's area and the duration of piano training was assessed. First, subtraction of the averaged oxy-Hb value in the familiar condition from that in the unfamiliar condition was individually calculated as an index that shows the sensitivity to action hierarchy. Then, the correlation between such contrast and the duration of piano training was evaluated using a Spearman's rank correlation test. The significance level was set to 5%.

## Results

**Figure 2:**
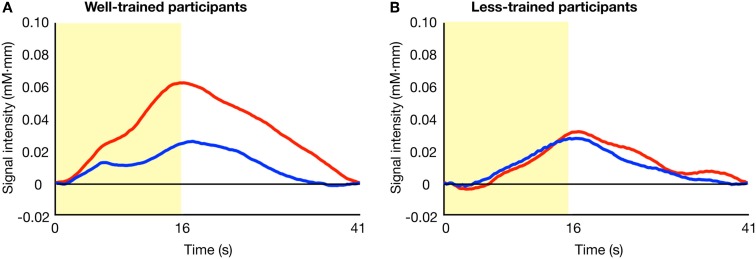
**Average time series data of the oxy-Hb concentration changes after stimulus onset**. The results of the familiar (blue lines) and unfamiliar (red lines) conditions for well-trained **(A)** and less-trained **(B)** participants are shown. The yellow area indicates the stimulation period.

Figure 2 shows the average activity of Broca's area across time in the familiar and unfamiliar conditions. In the trained group (A), a difference in the patterns of signal change was evident between the two conditions. However, such a contrast was not observed in the less-trained group (B).

**Figure 3:**
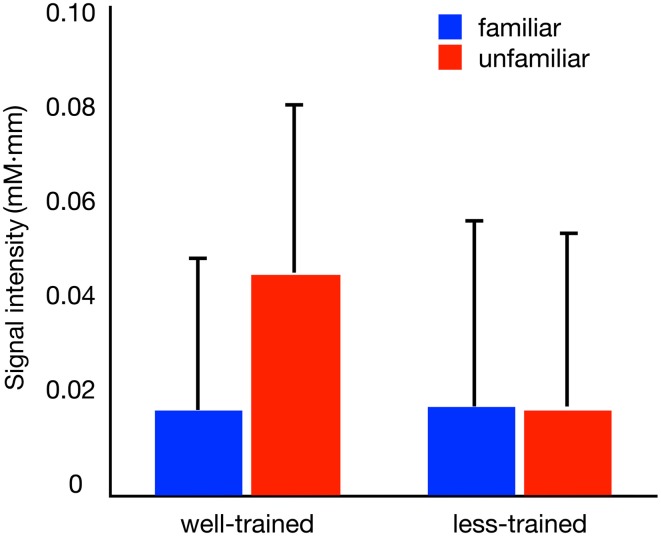
**Average oxy-Hb concentration changes within the peri-stimulation period**. The results (+1 *SD*) of the familiar (blue bars) and unfamiliar (red bars) conditions for well-trained and less-trained participants are shown.

The activation of Broca's area within the peristimulus period is shown in Figure 3, where the mean signal intensities for individual participants are compared between conditions. In the well-trained group (left), statistical analysis revealed a significant difference in the signal intensities between conditions [familiar condition (mean ± SD): 0.018 ± 0.031 mM·mm, unfamiliar condition: 0.045 ± 0.035 mM·mm] [paired *t*-test, *t*_(9)_ = 3.167, *p* = 0.011]. Contrary to the well-trained group, the less-trained group (right) showed no significant difference in signal intensities between conditions [familiar condition: 0.018 ± 0.038 mM·mm, unfamiliar condition: 0.018 ± 0.036 mM·mm] [paired *t*-test, *t*_(9)_ = 0.076, *p* = 0.941]. Thus, the involvement of Broca's area in the hierarchical processing of observed action was observed in the well-trained participants.

**Figure 4:**
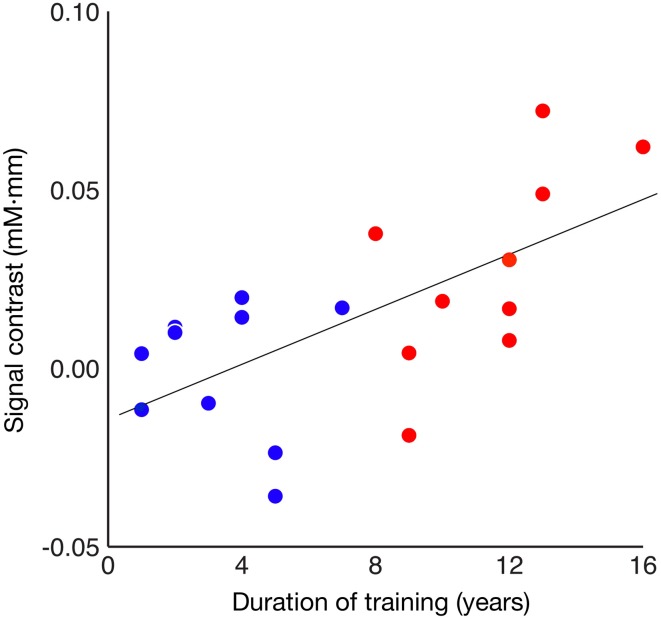
**Correlation between signal contrast between familiar and unfamiliar conditions and duration of piano training**. Red and blue dots represent the results of well-trained and less-trained participants, respectively.

The relationship between the degree of such involvement of Broca's area and the duration of piano training were then assessed. A correlation analysis of the entire population revealed that the between-condition difference in mean signal intensity strongly correlated with the duration of piano training [Figure 4; Spearman's rank correlation test, *rho*_(18)_ = 0.593, *p* = 0.006]. When a correlation analysis was applied to individual groups, however, no significant correlation was found both in the well-trained group (red dots; α = 0.05/2, *rho*_(8)_ = 0.654, *p* = 0.040) or the less-trained group (blue dots; α = 0.05/2, *rho*_(8)_ = 0.092, *p* = 0.800).

## Discussion

### General conclusion

The primary goal of the present study was to determine the role of Broca's area in the hierarchical processing of observed action. To address this issue, well-trained and less-trained participants were exposed to silent hand motions that played familiar and unfamiliar melodies. A key finding was that well-trained participants showed increased activation in Broca's area in response to hand motions associated with unfamiliar rather than familiar melodies. Thus, the current data indicate that this region plays an essential role in the hierarchical processing of an observed action. Previous fMRI and magnetic stimulation studies that showed that Broca's area is involved in hierarchical sequence of action planning (Koechlin and Jubault, 2006; Clerget et al., 2011, 2013) may support the current findings.

Additionally, a correlation analysis of the entire population exhibited that the Broca's area of participants who had longer piano training experience exhibited a stronger sensitivity to the hierarchical structure of observed action. However, because there was no such correlation within each participant group, sensitivity to action hierarchy may not develop monotonically as a function of the duration of piano training. Rather, the current results imply that Broca's area becomes sensitive to perceived motor hierarchy only after sufficient motor training.

### Was the hand motion meaningless to the less-trained participants?

In the current study, the less-trained participants showed no significant difference in the activation of Broca's area between the familiar and unfamiliar melodies. It has been shown that the activation of Broca's area is associated with an action that is meaningful or possible to the observer (Decety et al., 1997; Grèzes et al., 1998; Costantini et al., 2005; Wakita and Hiraishi, 2011). Therefore, it may be assumed that the observed actions were meaningless to the less-trained participants. The debriefing after the recording session revealed that neither the well-trained nor less-trained participants could report what the unfamiliar melodies were like, but both well-trained and less-trained participants were able to identify the familiar melodies by the observation of key-touch. Therefore, the task performance was comparable between both groups of participants, and the hand movements featuring familiar and unfamiliar melodies were meaningful actions for both groups of participants. How can the difference in the activation of Broca's area between well-trained and less-trained participants explained?

### Processing of observed action in broca's area of well-trained and less-trained participants

The current study implied that Broca's area is also responsible for the analysis of hierarchical structure in a wide range of functional domains. Therefore, it must be significant to survey how native and non-native languages are processed in Broca's area prior to considering the effects of motor expertise on the activity of this area.

Rüschemeyer et al. (2006) showed that BA 44 of a native speaker becomes active only when the processing of a structurally complex sentence is demanded; BA 44 is less active when processing a simple sentence. However, BA 44 of non-native speakers is consistently active, regardless of the degree of complexity of sentences (see also Rüschemeyer et al., 2005; Yokoyama et al., 2006). Moreover, Jeon and Friederici (2013) demonstrated that the syntactical analysis of complex non-native sentence recruited BA 47. Thus, only BA 44 of native speakers showed complexity-dependent activation. However, the less-proficient syntactical processing of non-native speakers is compensated for by the semantic information in the sentences.

When well-trained native and less-trained foreign language processing in Broca's area is taken into account, the difference in the activation of this area between well-trained and less-trained participants may be plausibly explained. The current results indicated that Broca's area in well-trained participants was sensitive to the hierarchical organization of hand motion. This effect was likely due to the extensive piano experience of the participant and the concurrent acquisition of neuronal mechanisms in Broca's area that represent action hierarchy. Based on the findings by Rüschemeyer et al. (2006), as well as Jeon and Friederici (2013), it is conceivable that Broca's area is not necessarily activated by processing a simple hand motion action. Rather, Broca's area may come into play only when processing an unfamiliar hand motion sequence.

However, Broca's area in less-trained participants was not sensitive to the hierarchical organization of hand motion. Again, based on the aforementioned linguistic studies, less-trained participants most likely employed neuronal mechanisms that identified “melodies” by relying on the meaning of the melody [mediated by BA 47 (Platel et al., 1997)] rather than by relying on the hierarchical organization of hand motions. Consequently, the appearance of hand motion may induce an equivalent degree of activation in Broca's area regardless of the familiarity of the stimulus. Future studies may compare the activation from the posterior part of the inferior frontal region (BAs 44 and 45) and the anterior part of the inferior frontal region (BAs 45 and 47) to investigate the effects of expertise on the development of the representation of action hierarchy in Broca's area.

### Role of broca's area as a domain-general hierarchical processor

Previous studies have reported that familiar body movements evoked increased activation of several brain regions of the mirror neuron system (MNS) than unfamiliar movements (see Rizzolatti and Craighero, 2004; Fadiga et al., 2005 for review). For instance, Calvo-Merino et al. (2005) found a stronger recruitment of MNS including left ventral premotor cortex (vPM) close to Broca's area and bilateral intraparietal sulcus in expert ballet and capoeira dancers for the observation of the dance style of a familiar genre when compared with the unfamiliar genre. The authors argued that the activation of MNS reflected an action resonance processes. However, as far as observation-related brain activation is explained by an uncertain notion, i.e., “action resonance,” we do not currently understand the differential processing of information within and between brain regions among MNS.

Alternatively, considering the shared function of Broca's area in a wide range of functional domains must be helpful to understand the role of this area in MNS. Musso et al. (2003) indicated that Broca's area is centrally involved in the processing of grammatical rules. They trained a native speaker of German to learn the grammatical rules of a non-native language with different grammatical rules (e.g., Italian and Japanese). Consequently, they found a significant correlation between the degree of activation of Broca's area and the performance of the judgment of syntactical correctness of language. Thus, Broca's area became active only when regularity of hierarchical structures was successfully abstracted from the sentence.

Given that the acquisition of hierarchical structures is an essential factor for Broca's area to be active, the vPM activation in Calvo-Merino et al. (2005) may be explained in terms of the ability to process action hierarchy. Because classical ballet and capoeira do not share poses and motion sequences with each other, it is plausible to assume that ballet dancers could abstract the regularity of a ballet dance sequence but could not abstract action hierarchy from capoeira. Consequently, the inferior frontal region of ballet dancers may have been more thoroughly recruited for the observation of ballet compared with capoeira and vice versa. Thus, if the notion that Broca's area process hierarchical structures in a wide range of functional domains is correct, this may provide insights into the neural basis of action understanding.

In conclusion, Broca's area was shown to be necessary for the hierarchical processing of observed action. Therefore, the notion that hierarchical processing in Broca's area is a common function shared between the language and music domains may help to explain the role of Broca's area in action perception. In support of these findings, previous studies have reported that the effect of expertise in one domain leads to the improvements in tasks of untrained domains such as enhanced linguistic performance after musical training (Moreno et al., 2009; Strait et al., 2012; François et al., 2013). The effect of auditory rhythm on motor functioning has also been suggested (de Dreu et al., 2012; Hardy and LaGasse, 2013). In summary, the notion of Broca's area as a domain-general hierarchy processor may provide insight into the beneficial transfer across language, music and action abilities.

### Conflict of interest statement

The author declares that the research was conducted in the absence of any commercial or financial relationships that could be construed as a potential conflict of interest.

## Supplementary material

Supplementary Video 1 **Example of a familiar stimulus**. This video shows a familiar stimulus generated from “Mary Had a Little Lamb.”

Supplementary Video 2 **Example of an unfamiliar stimulus**. This video shows an unfamiliar stimulus generated from “Mary Had a Little Lamb.”
